# Genotype-Phenotype Correlations in a Mountain Population Community with High Prevalence of Wilson’s Disease: Genetic and Clinical Homogeneity

**DOI:** 10.1371/journal.pone.0098520

**Published:** 2014-06-04

**Authors:** Relu Cocoş, Alina Şendroiu, Sorina Schipor, Laurenţiu Camil Bohîlţea, Ionuţ Şendroiu, Florina Raicu

**Affiliations:** 1 Chair of Medical Genetics, “Carol Davila” University of Medicine and Pharmacy, Bucharest, Romania; 2 Francisc I. Rainer Anthropological Research Institute, Romanian Academy, Bucharest, Romania; 3 Genome Life Research Centre, Bucharest, Romania; 4 National Institute of Endocrinology “C. I. Parhon”, Bucharest, Romania; 5 Family Medical Centre, Rucar, Romania; 6 Sf. Pantelimon Clinical Emergency Hospital, Bucharest, Romania; Pasteur Institute of Lille, France

## Abstract

Wilson’s disease is an autosomal recessive disorder caused by more than 500 mutations in ATP7B gene presenting considerably clinical manifestations heterogeneity even in patients with a particular mutation. Previous findings suggested a potential role of additional genetic modifiers and environment factors on phenotypic expression among the affected patients. We conducted clinical and genetic investigations to perform genotype-phenotype correlation in two large families living in a socio-culturally isolated community with the highest prevalence of Wilson’s disease ever reported of 1∶1130. Sequencing of ATP7B gene in seven affected individuals and 43 family members identified a common compound heterozygous genotype, H1069Q/M769H-fs, in five symptomatic and two asymptomatic patients and detected the presence of two out of seven identified single nucleotide polymorphisms in all affected patients. Symptomatic patients had similar clinical phenotype and age at onset (18±1 years) showing dysarthria and dysphagia as common clinical features at the time of diagnosis. Moreover, all symptomatic patients presented Kayser-Fleischer rings and lack of dystonia accompanied by unfavourable clinical outcomes. Our findings add value for understanding of genotype-phenotype correlations in Wilson’s disease based on a multifamily study in an isolated population with high extent of genetic and environmental homogeneity as opposed to majority of reports. We observed an equal influence of presumed other genetic modifiers and environmental factors on clinical presentation and age at onset of Wilson’s disease in patients with a particular genotype. These data provide valuable inferences that could be applied for predicting clinical management in asymptomatic patients in such communities.

## Introduction

Wilson’s Disease (WD, OMIM #277900) is an autosomal recessive disorder of copper metabolism caused by mutations in the responsible gene, ATP7B, that codes for a membrane-bound copper-transporting P-type ATPase [1], [2], [3]. The ATP7B gene is located on chromosome 13 and has 21 exons spanning a DNA region of about 100 kb [4], [5], [6]. Over 500 mutations within the ATP gene have been identified along the whole length of the entire coding region and also in promoter and intronic regions (http://www.wilsondisease.med.ualberta.ca/database.asp). The worldwide prevalence of WD is estimated at one in 30000 and one in 100000 in most populations [7], [8], with a carrier frequency of 1 in 90 to 122 [9], [7]. The highest prevalence of WD was reported in the Sardinian (1∶7000) and Gran Canaria Island (1∶2600) populations due to inbreeding and founder effects [10], [11]. The frequency and distribution of ATP7B mutations in Romanian WD patients are not known precisely [12], [13].

The diagnosis of WD is made by clinical symptomatology in conjunction with biochemical, histological, imagistic data as established by Scheinber and Sternlieb [14], [15], [16] and/or genetic testing. Phenotypic classification could be realized using the WD classification scheme proposed by Ferenci *et al* [17]. Due to the wide range of clinical and biochemical features, Wilson’s disease is difficult to characterize clinically.

The clinical architecture of Wilson’s disease results from interactions between ATP7B and a spectrum of other genetic modifiers, environmental or lifestyle and stochastic factors that could have a degree of population and geographic specificity.

Copper accumulation can affect many organs especially brain or liver function generating diverse clinical presentations. Hepatic manifestations can range from asymptomatic liver and spleen enlargement to acute liver failure and cirrhosis, while neuropsychiatric manifestations can range from tremor, dysarthria, dystonia and cognitive dysfunction with or without hepatic presentation.

No definite genotype-phenotype correlations have been established so far due to the allelic heterogeneity and the rareness of the disease. However, few papers have suggested possible relationships between age at onset or type of presentation and a specific genotype [18], [19], [20], [21].

Genealogic investigation allowed us to cluster two large families in a common multigenerational pedigree in a socio-culturally isolated mountain community with the highest WD prevalence ever reported. As outlined by other reports, an isolated community is a powerful resource for genetic studies as a consequence of limited genetic heterogeneity of their inhabitants who are more likely to share additional common genetic modifier factors and have similar eating habits that could increase the chances of finding subjects with the same ATP7B genotype and performing genotype-phenotype correlations [22], [23].

Here, we conducted a genetic analysis of seven WD patients and 43 family members in two large families spanning six generations and focused on a detailed evaluation of genotype-phenotype correlation. Our results could support the hypothesis of equal effect on WD clinical presentation and age at onset of genetic modifier factors in such populations.

## Subjects and Methods

**Figure 1:**
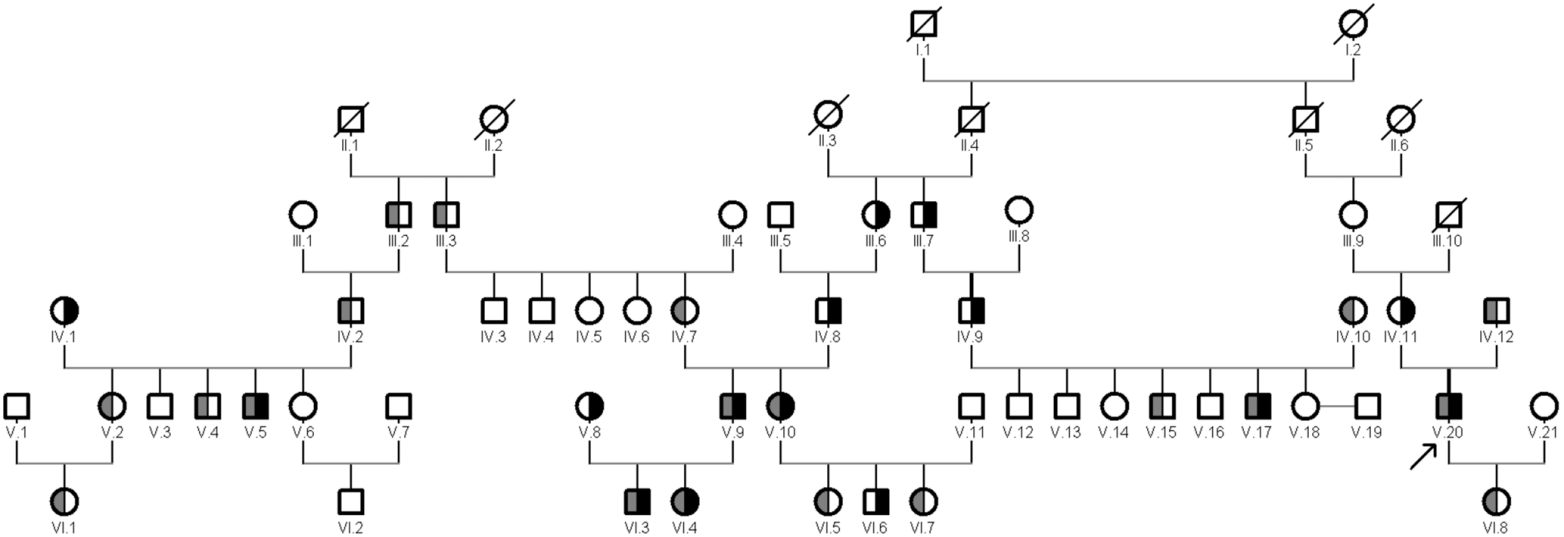
Pedigree and genetic analyses of the two large families. Genetic analyses were performed on all individuals indicated by filled, divided and open symbols. Pedigree symbols: slashed symbol, deceased individual; open symbol, unaffected individual; divided gray symbol, carrier for M769H-fs mutation; divided black symbol, carrier for H1069Q mutation; filled symbol, affected individual with compound heterozygous genotype. A filled arrowhead indicates proband. Roman numbers indicate generations.

We studied two large families, which spanned six generations consisting of 50 living members, of which 7 were affected by WD. We were able to link these two families based on information provided by relatives of patients in an extensive multigenerational pedigree sharing 4 unique family names (Figure 1). No consanguinity was recorded among parents and the families are inter-connected only through the last three generations. The proband was a 59-year-old male who underwent clinical assessment and was diagnosed with WD at the age of 19. The pedigree has been modified to protect the anonymity of the families. Informed written consent was obtained in accordance with protocols approved by the “Carol Davila” University of Medicine and Pharmacy’s ethical committee. These families are located in Rucar in a mountain region having a current population size estimated at 6200 with a possible high level of consanguinity in the past.

### Clinical Diagnosis

We initially based the diagnosis of affected members on neurological clinical symptomatology like the presence of a Kayser-Fleischer (K-F) ring using slit-lamp examination, typical neurological symptoms, and hepatic features including symptoms of acute, chronic cirrhosis and fulminant liver failure, and the presence of conventional biochemical markers like low serum ceruloplasmin (<20 mg/dL) and elevated baseline 24-hour urinary copper excretion (baseline levels >100 µg/24 h), (Table 1). Due to parental refusal or religious convictions and since most of the patients were neurologic, liver biopsy was not carried out as a diagnostic measure. We performed a second complete clinical evaluation complemented with liver biochemical and genetic analysis in this study for all affected and asymptomatic subjects.

**Table 1:** Clinical and laboratory findings of WD patients.

			Clinical presentation at diagnosis			Laboratory Findings at diagnosis			
Patient No	Age at onset (y), sex	Age at diagnosis (y)	Hepatic	Neurological	K-F ring	Serum CP (mg/dL)	Urinary Cu (µg/day)	ALT (U/L)	AST (U/L)
V.5	17, M	17	−	+	+	3.5	340	35	40
V.10	18, M	18	−	+	+	0.9	612	20	19
V.9	18, F	19	−	+	+	2.6	1019	27	20
VI.3	6, M	6	−	−	−	0.4	70.5	278	133
VI.4	7, F	7	−	−	−	0.1	210	339	143
V.17	19, M	19	−	+	+	1.2	413	NA	NA
V.20	19, M	20	−	+	+	2.3	507	NA	NA

Abbreviation and Notes: M, male, F, female; y, years; NA, Not available; K-F, Kayser-Fleischer; CP, Ceruloplasmin; Cu, Copper, ALT, Alanine Transaminase; ASP, Aspartate Transaminase.

Serum CP was measured by immunoturbimetric test. Serum CP normal values are 20–60 mg/dL.

Normal urinary copper values is less than 100 µg/day.

Normal ranges of liver enzymes are: ALT (10–45 Units/L) and AST (15–47 Units/L).

Patient numbering is represented as indicated in the pedigree.

### Mutation Sequence Analysis

We extracted genomic DNA from whole blood in EDTA using PureLink Genomic DNA Mini kit (Invitrogen, USA). We performed mutation analysis on PCR amplified DNA for the entire 21 coding exons, their exon-intron boundaries and 600 base pair of promoter with primers previously reported [24], [25] and other primers that are available on request using AmpliTaq Gold polymerase (Applied Biosystems, USA) according standard protocols. We purified PCR products with QIAquick PCR purification kit (Hilden, Germany) and sequenced with the Big Dye Terminator v3.1 Cycle Sequencing kit using ABI PRISM 310 and ABI 3130XL Genetic Analyzers (Applied Biosystems, USA). Analyses of ATP7B DNA sequencing data were performed using the ABI PRISM DNA Sequencing Analysis Software, Version 3.7. The sequences were aligned and compared with the revised Cambridge Reference Sequence rCRS (NM_000053, NCB), using the SeqScape Software Version 2.5. We confirmed the detected mutations and SNPs on both sequencing platforms in forward and reverse directions and compared with the revised Cambridge Reference Sequence rCRS using the SeqScape Software, Version 2.5 and MEGA5.

## Results

### Sequencing Results

Sequencing results revealed two mutations, c.3207C>A (p.His1069Gln) and c.2304insC (p.Met769His-fs), and seven additional single nucleotide polymorphisms (SNPs) in exons/introns: exon 2, c.1216 T>G (p.Ser406Ala); exon 3, c.1366G>C (p.Val456Leu); exon 10, c.2495A>G (p.Lys832Arg); exon 12, c.2855G>A (p.Arg952Lys); intron 13, c.2866–13G>C; exon 16, c.3419C>T (p.Val1140Ala) and intron 18, c.3903+6C>T in seven members affected by WD outlined in a common pedigree. Of these two mutations, one is a missense mutation, H1069Q, located in exon 14 and the other is a frameshift mutation, M769H-fs, lying in exon 8. The mutations described herein, p.H1069Q and p.M769H-fs, alter ATP loop and Tm 4 domain, respectively.

These two mutations are represented as compound heterozygous in the proband (V.20) and other four symptomatic patients in two families outlined in a large apparently non-consanguineous pedigree in a socio-culturally isolated community with high prevalence of WD (Figure 1). By direct sequencing of ATP7B gene we identified the same compound heterozygous genotype, H1069Q/M769H-fs, in both asymptomatic children (VI.3 and VI.4) of parents (V.8 and V.9). The mutation M769H-fs is represented as heterozygous in 13 unaffected members and the mutation H1069Q is represented as heterozygous in 8 unaffected members in the pedigree.

We screened a total of 43 family members for the identified mutations and SNPs. The discovered SNPs, previously reported [26], [27], [28], [29], [30], [31], [32] were tested in 102 healthy controls with their frequencies presented in Table 2.

**Table 2:** The SNPs of the ATP7B gene in healthy control group.

Exon/intron	Nucleotide	Amino acid	Protein domain	Type	SNP	Allelefrequency (%)
2	c.1216 T>G	p.Ser406Ala	Cu_4_	Missense	Known	39
3	c.1366G>C	p.Val456Leu	Cu_5_	Missense	Known	42
10	c.2495A>G	p.Lys832Arg	A-domain/Td	Missense	Known	65
12	c.2855G>A	p.Arg952Lys	TM5	Missense	Known	16
Intron 13	c.2866–13G>C	-	-	-	Known	46
16	c3419C>T	p.Val1140Ala	ATP loop	Missense	Known	31
Intron 18	c.3903+6C>T	-	-	-	Known	26

Abbreviation and Notes: SNP, single nucleotide polymorphism. Nucleotide numbering refers to the cDNA according GenBank Accession number NM000053, where the first nucleotide of ATG translation codon is considered nt +1. Total number of alleles was 204.

In the pedigree, two or more SNPs occurred simultaneously at different haplotype combinations among affected patients and healthy members. Two of the seven SNPs, c.2495A>G and c.3419C>T, were present in all symptomatic and asymptomatic patients.

### Clinical Data

Patient V.20, the proband of pedigree, was initially diagnosed with WD at the age of 20 in 1974, although he first exhibited neurological signs including dysarthria in one hand, mild dysphagia and malaise at the age of 19 without seeking medical advice. He was started on D-penicillamine treatment at age of 21 years due to the unavailability of the drug in Romania at the time of his diagnosis. The patient responded to the treatment and his conditions improved slightly. Ophthalmologic examination revealed bilateral Kayser-Fleischer rings. After the onset of the disease, he gradually developed neuropsychiatric complications including advanced dysarthria, drooling, postural tremor and instability, chorea, parkinsonism and cognitive manifestations like slowness of thinking and executive dysfunction. Decompensated liver cirrhosis was present with Child-Pugh score B at the age of 36 years with mild ascites, jaundice, coagulopathy, hyperbilirubinemia and hypoalbuminemia.

Patient V.5 was initially admitted to hospitalization at the age of 17 years in 2000 with signs of liver failure including subfebrile temperature, jaundice, fatigue and vomiting. The pathological findings were initially interpreted as a presumed hepatic viral infection but investigation of his nervous system revealed clinical symptoms including dysarthria, mild dysphagia and malaises. His serum ceruloplasmin and his 24-hours urinary copper levels were in pathological range (Table 1). In addition, the presence of the pathognomonic clinical sign, presence of the Kayser-Fleischer rings in both eyes, was confirmed by ophthalmoscopic examination by slit lamp examination. He was started on low D-penicillamine (250 mg/day) dose and continued with slowly increasing doses that resulted in a modest improvement but was diagnosed 2 years later with compensated liver cirrhosis with Child-Pugh score A. In 2013, he was diagnosed with acute myeloid leukemia.

Patient V.17 was diagnosed with WD in 1987 at the age of 19, presenting with mild dysphagia, mild dysarthria and headache. Kayser-Fleischer rings initially unobserved were subsequently detected bilaterally by slit lamp examination. His serum ceruloplasmin and 24-hours urinary copper levels are presented in Table 1. He has been treated with D-pencillamine with incremental doses ever since, with initial slight improvement in his condition, but subsequent worsening the disease. Signs of mild hepatic manifestations without jaundice and haemolytic anemia presented later. The patient has developed major neurological features including pronounced dysphagia and dysarthria, drooling, unsteady gait and psychiatric manifestations like agoraphobia, slowness of thinking and mood disturbances.

Patient V.9 was diagnosed with WD at the age of 18 presenting with progressive neurological symptoms, including dysarthria, dysphagia without impairment of motor skills. His ceruloplasmin levels were low (<2.6 mg/dL) and his 24-hours urinary copper high (>1019 µg/day). Ophthalmologic examination revealed bilateral Kayser-Fleischer rings. Response was initially positive to D-penicillamine therapy. Five years later, abdominal ultrasonography examination detected a mild echogenicity in the liver. Dysarthria became pronounced over the years with subsequently developed body bradykinesia, resting and postural tremor.

Both children of this patient (VI.3 and VI.4), were clinically asymptomatic, but were diagnosed with WD by biochemical tests at the age of 6 and 7, respectively. An apparent pseudo-dominat inheritance with two consecutive generations affected by Wilson’s disease could be seen in parents and their children in this family.

The children’s neurological tests were normal, however laboratory findings were clearly abnormal consisting of increased 24 h urinary copper values, decreased serum ceruloplasmin levels and high levels of aspartate transaminase (AST) and alanine transaminase (ALT), (Table 1). Preventive treatment with D-pencillamine was started for both children once they were diagnosed.

For patient V.10, the sister of V.9 (affected), symptoms began when she was 19 years old. She indicated that the symptoms started insidiously when she was 18. At the time of diagnosis, signs of neuropsychiatric disturbances without liver presentation were present including dysphagia, nystagmus, and dysarthria, lacking signs of tremor and postural instability, and mild cognitive impairment like difficulties in school performance. Kayser-Fleischer rings were present on both eyes. The response to D-penicillamine therapy was adequate, showing slight improvement, but was discontinued during her pregnancies. In a very short period of time, she suffered a gradual psychiatric deterioration resulting in depression, personality changes and behavioral disturbances associated with advanced neurological clinical features including body bradykinesia, tremor and postural instability.

Unaffected family members of all WD subjects had a full evaluation and were found normal. Their physical examination, serum ceruloplasmin, serum copper, ALT, AST and 24 h urine copper levels were normal.

Severe neurological deterioration was observed in all symptomatic patients without relevant side effects. Three of the symptomatic patients with initial neurological signs, patients V10, V.7 and V.15, developed a parallel deterioration of hepatic function while under treatment with evidence of cirrhosis, either compensated in patient V.10 or decompensated in patient V.20, whereas the other two remained only neurologically symptomatic with stable liver function. Both asymptomatic patients showed no normalization of biochemical tests under drug therapy.

## Discussion

The extensive variation in hepatic and neurological presentations in affected patients carrying an identical genotype in different families or within the same family is one of the most intriguing aspects of Wilson’s disease [2], [19], [33]. It is unknown why a particular genotype is not associated to a specific behavior of the disease, albeit some authors have tried to establish a correlation between the type of presentation, age at onset or clinical course and the presence of a specific mutation in heterozygous or homozygous state [20], [21], [34], [35].

Since the Middle Ages for more than 600 years wealthy families of shepherds in Rucar region practiced marriages between members of the same clan as a way of protecting the family inheritance. This marriage pattern changed only at the beginning of 20th century. As a result of this socio-cultural practice, a period of genetic isolation could have occurred definitely affecting the actual genetic structure of this population. Although the actual prevalence of consanguineous marriages is very low in Romanian population, a substantial level of consanguinity would have been inevitable in scattered mountain rural communities in the past. Isolation could still play a role in this region as a result of the absence of immigration generated by poor economic resources.

On the basis of the number of Wilson’s disease patients and births recorded between 1975–2012 we calculated the prevalence of the disease to be 1∶1130. This is the highest prevalence ever reported [10], [11], [36].

As summarized in our paper, there were significant similarities at the time of diagnosis with respect to clinical features and ages at onset. Pedigree analysis revealed an apparent pseudo-dominant inheritance case in which two consecutive generations presented family members with Wilson’s disease. A similar situation was reported by other studies in consanguineous or distant consanguineous families [24], [37], [38].

In most published papers, authors compared patients from distinct families that are homozygous for a particular mutation, while others compared homozygotes for the same type of mutation [19], [39].

As a result of identical age at onset and similar clinical presentation among all our symptomatic patients, we suggest a dominance effect of frameshift mutation p.M769H-fs over missense mutation p.H1069Q. Although the presence of an intermediate effect as previously suggested by Gromadzka *et al* [34] or the influence of other genetic factors could not be excluded.

The presence of a coexisting frameshift mutation, p.H1069Q, in compound heterozygous state in our patients was associated with lower age at onset that is in agreement with previously reported results regarding the ages at onset for p.H1069Q homozygotes and p.H1069Q/missense patients [39], [40].

Møller *et al* [41] classified mutations either as severe or moderate based on whether they cause clinical symptoms before or after the age of 20, assuming the disease severity defined by the age of onset is determined by the less severe of two mutations. In our compound heterozygote patients carrying two severe mutations according to Møller classification, p.H1069Q and p.M769H-fs, the age at presentation (18±1 years) fits exactly with the proposed algorithm. In contrast, Gupta *et al* [33] obtained contradictory data suggesting that the age of onset is established by the most severe from the two mutations.

Dysarthria and dysphagia, either mild or advanced, were the first common signs observed for all symptomatic WD patients, except for two asymptomatic children that could follow an identical clinical course without medication. Moreover, in all symptomatic patients Kayser-Fleischer rings were present without showing a very common neurological sign, namely, dystonia.

Several familial studies have shown that despite phenotypic variation, siblings present an identical clinical type or age at onset [42], [43] while a few authors observed no genotype-phenotype association even among the same homozygote or compound heterozygote genotype siblings or monozygotic twins [33], [44], [45].

In a recent paper, Chabik *et al* [43] reported results similar to our findings demonstrating a high intra-familial concordance of WD patients with a less predictability for neurological presentation. Furthermore, our study indicated a great clinical predictability even for neurological presentation by the presence of the same set of clinical features at the time of diagnosis and identical ages at onset (Table 3).

**Table 3:** Genotype-phenotype correlations found in patients with Wilson’s disease.

		ATP7B Genotype	Clinical symptoms at diagnosis		
Patient No	Mean age at onset 18±1 (y)	H1069Q/M769H-fs	Neurological presentation	Clinical findings	K-F ring
V.5	+	+	+	Dysarthria, dysphagia	+
V.10	+	+	+	Dysarthria, dysphagia, nystagmus	+
V.9	+	+	+	Dysarthria, dysphagia	+
V.17	+	+	+	Mild dysarthria, mild dysphagia	+
V.20	+	+	+	Dysarthria, mild dysphagia	+
VI.3	A	+	-	−	-
VI.4	A	+	-	−	-

Abbreviation and Notes: “−”, negative; “+”, positive; A, asymptomatic; y, years; K-F, Kayser-Fleischer;

These results demonstrate that the H1069Q/M769H-fs genotype is associated with common neurological symptoms at the time of diagnosis (dysarthria, dysphagia and K-F rings) and similar ages of onset, except for the two asymptomatic children that can have an identical clinical course without treatment. Patient numbering is represented as indicated in the pedigree.

For our symptomatic patients long-term follow-up revealed unfavourable outcomes with respect to the course of neuropsychiatric symptoms. Subsequently occurrence of other clinical features, neuropsychiatric and/or hepatic, in addition to initial common neurological signs and overall progressive clinical picture could be especially explained by the failure of medication, the time from diagnosis to treatment or periods of drug therapy discontinuation in some of patients. However, the implication of other presumed genetic factors could not be completely excluded. Development of acute myeloid leukemia, a very rare clinical feature in WD, was attributed by a single study to toxicity of D-penicillamine [46]. It seems unlikely that the occurrence of acute myeloid leukemia can be explained by the toxic effect of D-penicillamine for our patient repeatedly discontinued his medication use.

One of our most notable findings was the accelerated rate of disease progression of all symptomatic patients while under treatment with D-penicillamine. The progression of neuropsychiatric symptoms for all our patients while under treatment could be in concordance with Lee *et al* [47] finding that indicates a less favourable outcome for patients with neurological presentation compared to patients showing hepatic presentation.

The involvement of other presumed genetic modifiers factors such as ATOX1, COMMD1 and/or environmental factors could complicate the clear prediction of a specific phenotypic expression but their influence remains contentious as was suggested by other reports [47], [48], [49], [50]. Clinical heterogeneity in compound heterozygous patients influenced by the same environmental factors could not be entirely explained by the differing severity of particular alleles or other supplementary genetic modifiers but also by the additive effect of SNPs in ATP7B gene. Thus, two of the five exonic SNPs, c.2495A>G and c.3419C>T, were found present in all affected patients suggesting an identical additive effect of SNPs on phenotypic expression.

In conclusion, according to our results additional genetic modifiers and environmental factors would be expected to exert an equal influence on clinical picture and age at onset of WD in patients with a given genotype within the same or different families in relatively small isolated communities. Whereas a diverse effect would be expected some patients from diverse regions as a result of environmental and genetic heterogeneity as was demonstrated by other research.

Our patients offered a rare opportunity for assessing genotype-phenotype correlations considering the reduced worldwide availability of WD patients with a particular genotype living in isolated populations even if we could not draw definite conclusions. Our results suggest the use of genotypes to predict clinical manifestation and age at onset in asymptomatic patients in such communities.

## Supporting Information

Table S1 **The ATP7B mutations and SNPs detected in this study.**
(DOCX)

Table S2 **Electropherograms showing the mutations and polymorphisms in our study.**
(DOCX)
