# Supplementary material for: Genotype-Phenotype Correlations in a Mountain Population Community with High Prevalence of Wilson’s Disease: Genetic and Clinical Homogeneity
Source: PLoS One. 2014 Jun 4;9(6):e98520. doi: 10.1371/journal.pone.0098520 (PMC4045667; doi:10.1371/journal.pone.0098520)
Supplement: Table S1 — The ATP7B mutations and SNPs detected in this study. (DOCX) [file pone.0098520.s001.docx]

**Table S1** ATP7B mutations and SNPs detected in this study

| Exon/Intron | Mutation |  |  |  |
| --- | --- | --- | --- | --- |
|  | Nucleotide change | Sequence | Amino acid | Type |
|  |  |  |  |  |
| Mutations |  |  |  |  |
| 8 | c.2304insC | CCC**C**ATG | p.Met769His-fs | Frameshift |
| 14 | c.3207C>A | CA**C**>CA**A** | p.His1069Gln | Missense |
| Polymorphisms |  |  |  |  |
| 2 | c.1216 T>G | **T**CT>**G**CT | p.Ser406Ala | Missense |
| 3 | c.1366G>C | **G**TG>**C**TG | p.Val456Leu | Missense |
| 10 | c.2495A>G | A**A**G>A**G**G | p.Lys832Arg | Missense |
| 12 | c.2855G>A | A**G**A>A**A**G | p.Arg952Lys | Missense |
| Intron 13 | c.2866-13G>C | TCT**G**TCC>TCT**C**TCC | intronic | No change |
| 16 | c.3419C>T | G**C**C>G**T**C | p.Val1140Ala | Missense |
| Intron 18 | c.3903+6C>T | GAG**C**G>GAG**T**G | intronic | No change |

Abbreviation and Notes: GenBank Accession number NM000053. The first nucleotide of ATG translation codon is considered nt +1.
