# Supplementary material for: Genotype-Phenotype Correlations in a Mountain Population Community with High Prevalence of Wilson’s Disease: Genetic and Clinical Homogeneity
Source: PLoS One. 2014 Jun 4;9(6):e98520. doi: 10.1371/journal.pone.0098520 (PMC4045667; doi:10.1371/journal.pone.0098520)
Supplement: Table S2 — Electropherograms showing the mutations and polymorphisms in our study. (DOCX) [file pone.0098520.s002.docx]

| Mutations | |
| --- | --- |
| Exon 8, p.Met769His-fs, c.2304insC,  CCC**C**ATG | Exon 14, p.His1069Gln, c.3207C>A,  CA**C**>CA**A** |
|  |  |
| SNPs | |
| Exon 2, p.Ser406Ala, c.1216T>G  **T**CT>**G**CT | Exon 3, p.Val456Leu, c.1366G>C,  **G**TG>**C**TG |
|  |  |
| Exon 10, p.Lys832Arg, c.2495A>G,  A**A**G>A**G**G | Exon 12, p.Arg952Lys, c.2855G>A, A**G**A>A**A**G |
|  |  |
| Intron 13, c.2866-13G>C,  TCT**G**TCC>TCTT**C**TCC | Exon 16, p.Val1140Ala, c.3419C>T,  G**C**C>G**T**C |
|  |  |
| Intron 18, c.3903+6C>T,  GAG**C**G>GAC**T**G |  |
|  |  |


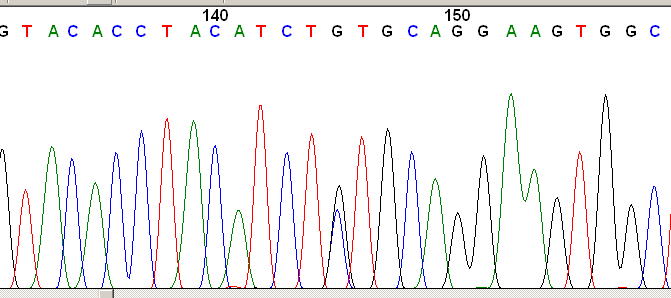

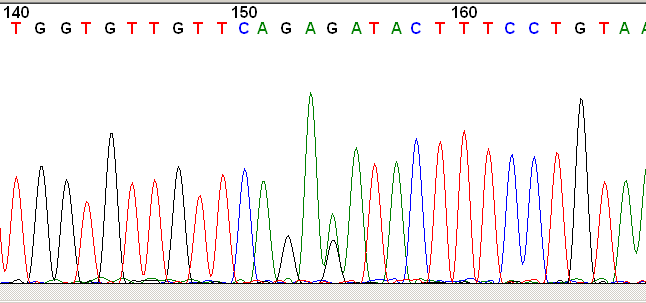


**Table S2** Electropherograms showing the mutations and polymorphisms in our study


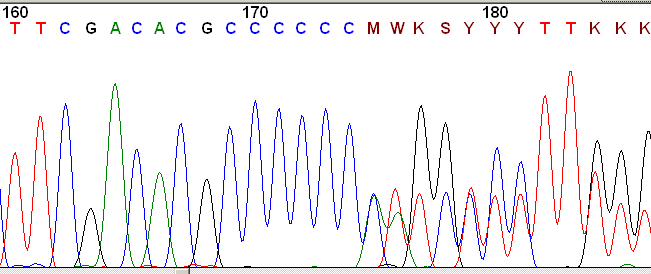

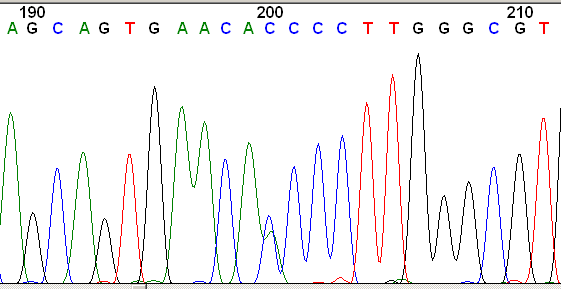

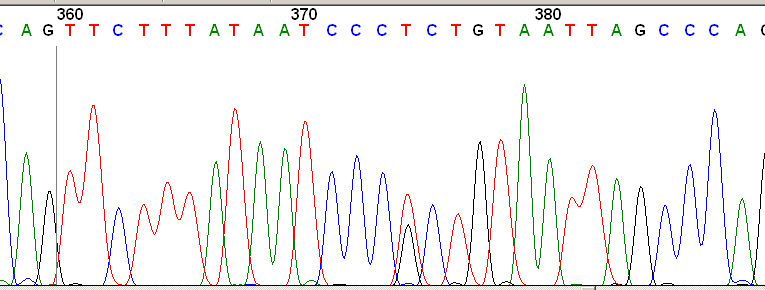

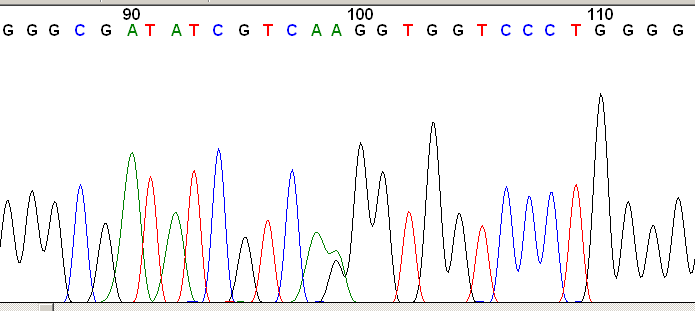

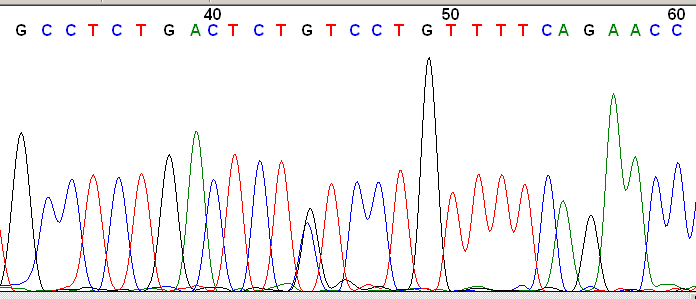

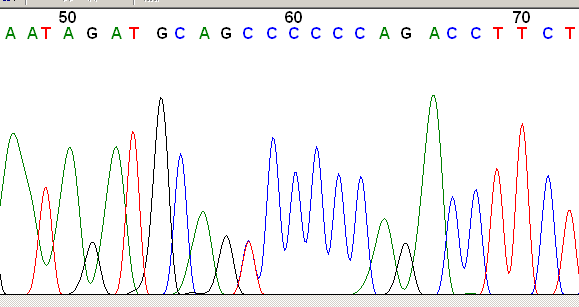

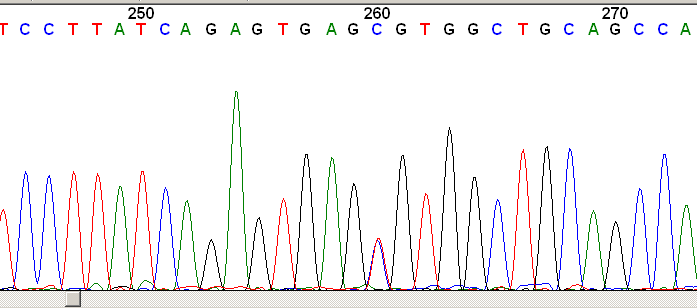


Note: The positions of the mutations and polymorphisms are indicated by arrows
